# Effects of Foot Somatosensory Training on Plantar Somatosensory Function

**DOI:** 10.7759/cureus.76399

**Published:** 2024-12-26

**Authors:** Junichi Suganuma, Yumi Ikeda, Kazuhiro Chidori

**Affiliations:** 1 Department of Physical Therapy, Graduate School of Human Health Sciences, Tokyo Metropolitan University, Tokyo, JPN; 2 Department of Physical Therapy, Faculty of Nursing and Rehabilitation, Chubu Gakuin University, Seki, JPN

**Keywords:** foot somatosensory training, somatosensory rehabilitation, spatial discrimination, tactile pressure sensitivity, two-point discrimination

## Abstract

Background

Declines in foot somatosensory function can negatively impact balance and daily activities, particularly in older adults and individuals with neurological conditions. Despite this, effective physical therapeutic interventions to improve foot sensory function are limited. This study assessed the effects of targeted foot somatosensory training on plantar sensory function in healthy young adults.

Methods

Thirty-three healthy young adults (mean age 21.2 ± 0.7 years) were randomly assigned to one of three groups: (1) Discrimination group, (2) Attention group, or (3) Control group. The Discrimination group performed spatial attention tasks to identify weight positions on an instability board; the Attention group focused on timing tasks; and the Control group watched a video. Tactile pressure sensitivity and two-point discrimination of the plantar surface were measured before and after the intervention. Statistical analyses included Wilcoxon signed-rank tests and two-way repeated measures of analysis of variance (ANOVA).

Results

No significant differences were found in tactile pressure sensitivity. However, two-point discrimination thresholds for the big toe and the ball of the little toe were significantly reduced in the Discrimination group, indicating enhanced sensitivity.

Conclusion

Foot sensory training, particularly through discrimination tasks, may effectively improve plantar sensory function, suggesting potential clinical applications in individuals with sensory impairments.

## Introduction

Sensory impairments are common following stroke, with prevalence rates reported at 47% for tactile sensation and two-point discrimination, 49% for proprioception, and 16% for tactile sensation on the nonparetic side. Approximately 80% of patients with stroke have sensory deficits in their limbs, highlighting the frequent occurrence of sensory impairments in many stroke cases [1]. However, despite the importance of assessing and treating sensory function, a cross-sectional survey of physical and occupational therapists found a lack of evidence-based expertise in addressing sensory function compared to motor function [2]. The reasons for this include the rarity of sensory impairment-focused treatments, prioritization of interventions for motor and basic functional abilities, and preference for compensatory strategies over direct recovery of sensory function. While interventions targeting motor function and fundamental movements are undeniably critical in clinical settings, improving sensory function may enhance motor abilities and basic functional capacity in cases where sensory impairment is a primary symptom. However, few treatment methods exist that directly address sensory function improvement.

Studies on somatosensory function interventions show that systematic sensory discrimination tasks (e.g., differentiation tasks) for the upper limbs can lead to improvements in somatosensory function [3], with patients with stroke reporting such tasks as rewarding [4]. For the lower limbs, interventions that induce stochastic resonance on the plantar surface using mechanical random noise stimulation have been reported to improve standing balance and tactile sensation in older individuals [5-7], those with diabetic peripheral neuropathy [8], and patients with stroke-induced hemiplegia [9]. However, these interventions are limited in clinical use because of the need for expensive and specialized equipment. Alternative approaches, such as tasks requiring the discrimination of five levels of sponge hardness while standing with eyes closed, have been shown to promote postural stability in young, healthy individuals [10], patients with stroke-induced hemiplegia [11], and older individuals with falls [12] after a set period of intervention. The mechanism for improved balance has been attributed to the enhanced somatosensory function of the plantar surface, which contributes to postural control stabilization. However, changes in plantar somatosensory function (e.g., tactile pressure sensitivity and two-point discrimination thresholds) before and after the task remain unclear.

Maintaining balance during standing relies on effective immediate somatosensory feedback loops from the foot area, necessitating coordinated ankle muscle activity and efficient ankle strategies. In addition to the discrimination of a single sensory modality such as sponge hardness (pressure), as shown in previous studies [10-12], incorporating comprehensive tasks that also involve joint position sense of the ankle may enhance plantar somatosensory function more effectively within a shorter timeframe.

In this study, we designed an intervention task involving sensory training for the foot by having participants place their feet in a seated position on an unstable board with weights positioned at four points (front, back, left, and right), which they were tasked with discriminating, along with feedback on accuracy to encourage coordinated muscle activity around the foot. We aimed to use this intervention to clarify whether improvements in plantar somatosensory function depend on the frequency of sensory stimulus input, attention to stimuli, or the combination of stimulus discrimination and focused attention across different training conditions.

## Materials and methods

Participants

Thirty-three healthy young adults (mean age 21.2 ± 0.7 years) were randomly assigned to one of three groups of 11 participants each, using a random number table generated in Microsoft Excel (Microsoft® Corp., Redmond, WA, USA): Discrimination group, Attention group, and Control group. Exclusion criteria included any overt orthopedic disorders of the foot, history of neurological disorders, sensory impairment in the foot area, or foot pain. After screening, none of the participants met the exclusion criteria and were eligible for inclusion in the study. The sample size was calculated using G*Power software (Heinrich-Heine-Universität Düsseldorf, Düsseldorf, Germany). With an effect size of 0.5, a significance level (α) of 0.05, and a statistical power (1-β) of 0.8, the required sample size for repeated measures of analysis of variance (ANOVA) with three groups was estimated. Based on these parameters, a total sample size of 33 participants was determined to be sufficient.

This study was approved by the Research Ethics Committee of the Graduate School of Tokyo Metropolitan University (approval no.: 24811). Informed consent was obtained from all participants, both verbally and in writing. In accordance with the International Committee of Medical Journal Editors (ICMJE) guidelines, this study was registered in the UMIN Clinical Trials Registry (UMIN-CTR) prior to initiation, under the registration number UMIN000056146.

Procedures

**Figure 1:**
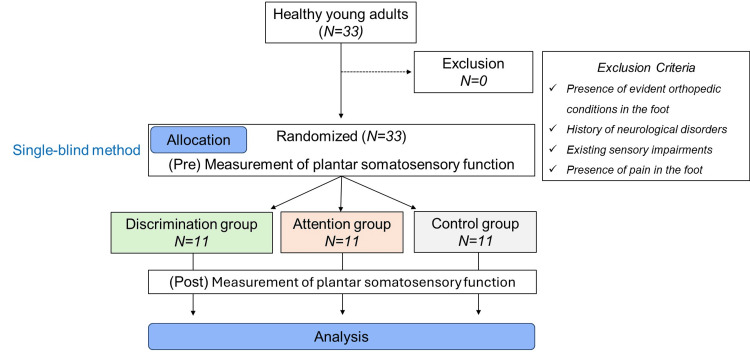
Flow diagram for randomized subject assignment in this study

In this single-blind randomized controlled trial, we randomly assigned the 33 participants to three groups: Discrimination, Attention, and Control, with 11 participants in each group (Figure 1). Randomization was performed using a random number table generated in Excel.

The specific details of each group’s intervention are outlined below:

Discrimination Group Protocol

**Figure 2:**
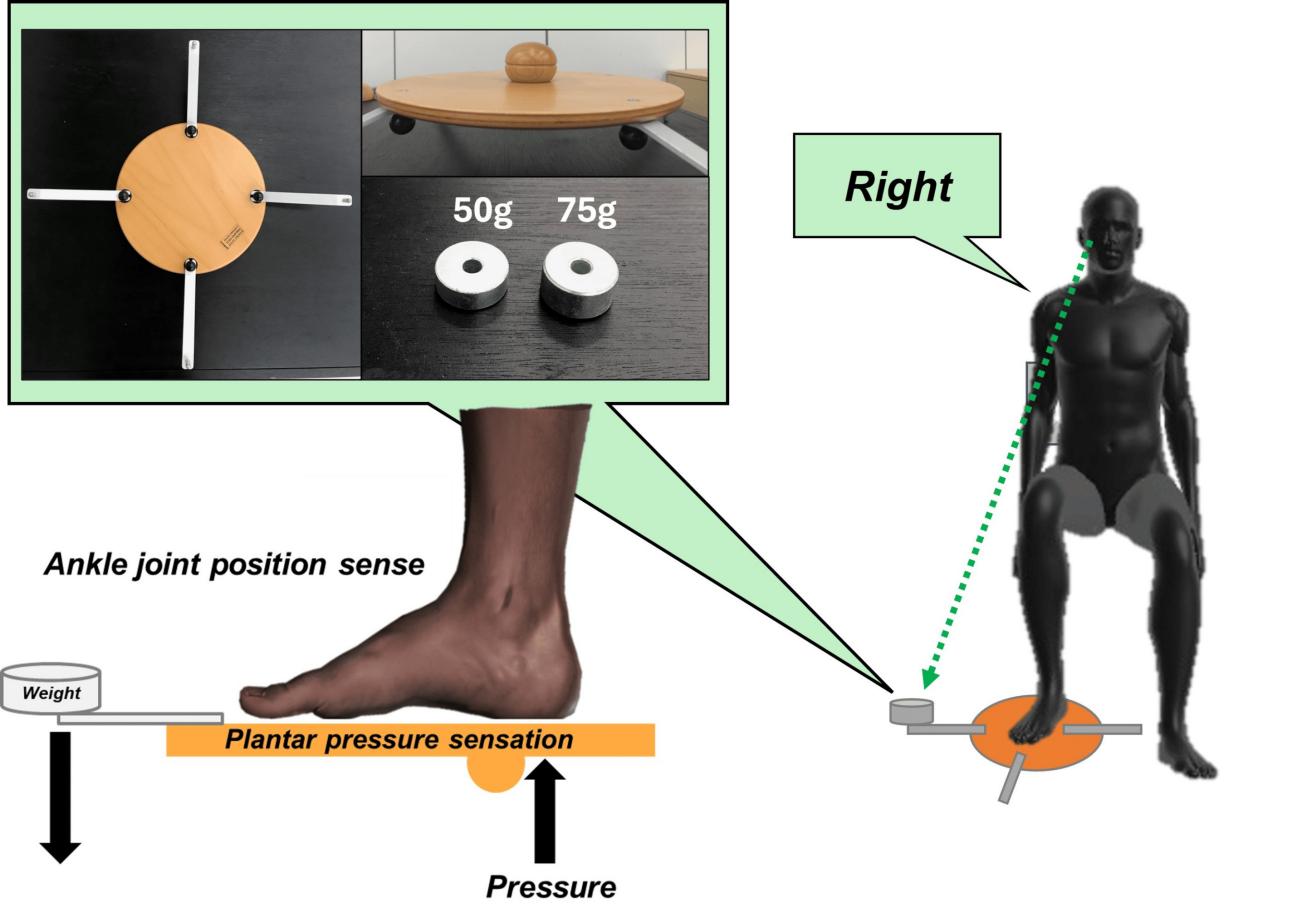
Overview of foot somatosensory training Image Credits: The image was created by the authors.

Participants in the Discrimination group performed a task involving barefoot placement of the sole on a multi-axial instability board (Cognitive Kit Set A2-2 series, manufactured by Fumagalli Co., Italy) while seated with eyes closed. A weight was randomly placed at one of four locations - front, back, left, or right - on the instability board, and the participants were asked to identify the weight’s position (Figure 2). The foot used for the task was defined as the dominant leg, which was identified as the leg used to kick the ball [13].

For the first task, a 100 g weight was used, and participants were required to correctly identify its position in 10 consecutive trials to proceed to the second task, which involved a 75 g weight. If successful, they moved on to the third task using a 50 g weight. The task was concluded if 10 consecutive correct responses were achieved in the final stage.

Feedback during the task was given as follows: correct answers received verbal confirmation, while incorrect answers prompted the participant to pause the task and open their eyes to visually confirm the weight’s position.

Attention Group Protocol

The Attention group followed the same posture and experimental setup as the Discrimination group but focused on timing rather than position. Participants were tasked with identifying the time when the weight was placed on the instability board and had to respond within approximately one second of placing the weight. Responses that were either too quick or slow were deemed incorrect.

For the first task, a 100 g weight was used, and participants needed to achieve 10 consecutive correct responses to advance to the second task using a 75 g weight. If participants completed 10 consecutive correct responses in the second task, they progressed to the third task with a 50 g weight. The task was concluded once the participants correctly responded in 10 consecutive trials in the final stage.

Feedback during the task was provided as follows: if the response was correct, verbal confirmation was provided, and the task continued. For incorrect responses, the task was paused, and the participants received feedback on the accuracy of their timing judgment.

The attention task required participants to react quickly to sensory stimuli, which engaged cognitive processes associated with sustained attention and selective attention. Previous studies have shown that tasks emphasizing reaction timing enhance the engagement of frontal-parietal networks, which are critical for attentional control and sensory integration [14,15]. While the attention task lacked spatial discrimination components, the focus on temporal precision likely activated neural pathways associated with attentional processing.

Control Group Protocol

Control group participants maintained the same posture and experimental setup as other groups but watched a medical drama for 20 minutes. To ensure that the video content was unfamiliar, each participant confirmed that this was their first viewing of the video. Afterward, they verbally recalled details about the characters and plot to confirm that they had actively engaged with the video. To standardize somatosensory input across the groups, a weight (one of the three types) was randomly placed on the multi-axial instability board every 30 seconds throughout the video session. This ensured that the foot sensory inputs of the Control group matched those of the Discrimination and Attention groups.

Measurement of plantar somatosensory function

**Figure 3:**
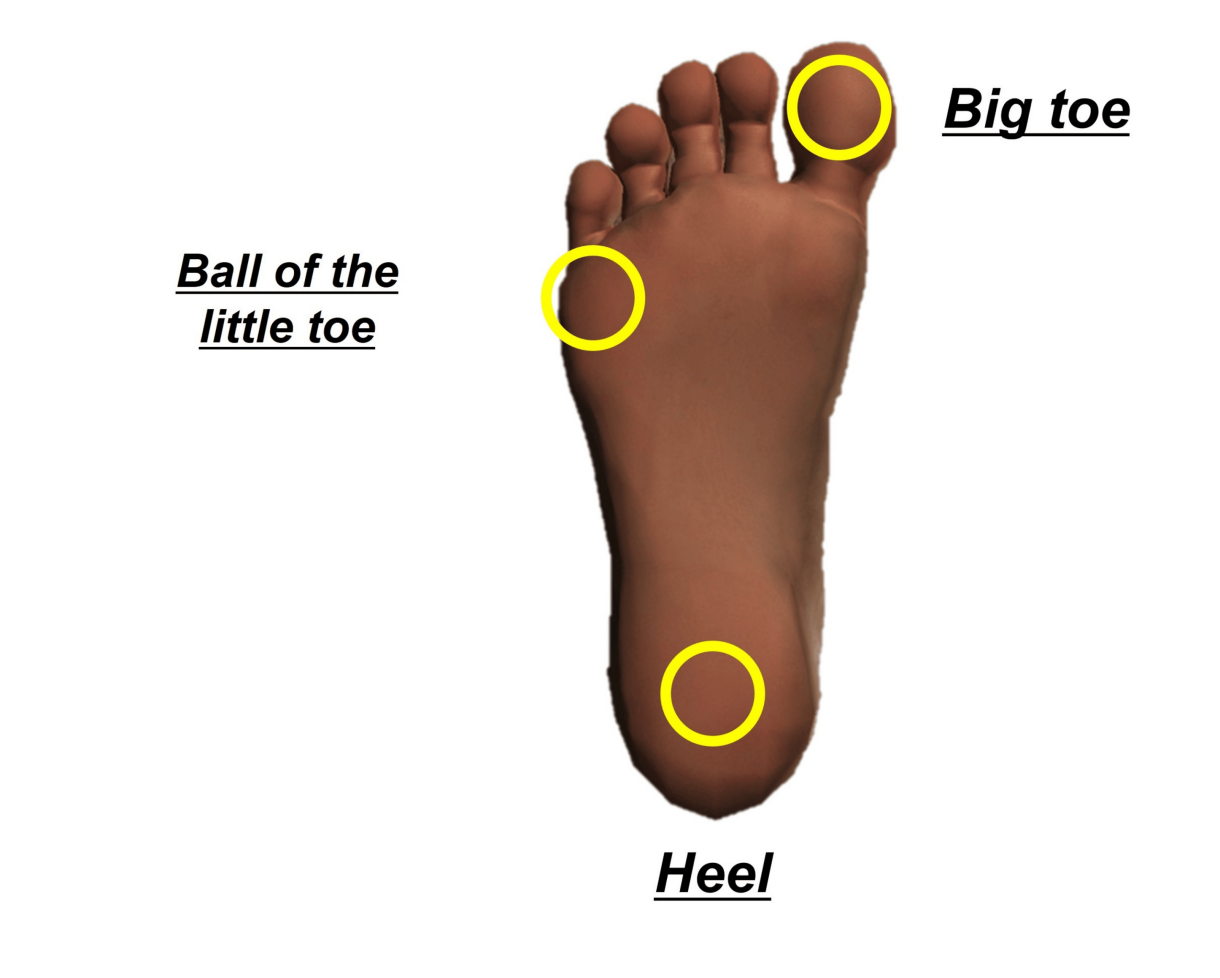
Measurement sites for plantar somatosensory function Image Credits: The image was created by the authors.

To evaluate the somatosensory function, plantar tactile pressure sensitivity and two-point discrimination thresholds (big toe, ball of the little toe, and central heel) were measured using 20 types of Semmes-Weinstein monofilaments and a Disk-Criminator (Marstock Instruments, Germany) (Figure 3). Sensory thresholds were compared pre- and postintervention.

The psychophysical staircase method was employed, starting with the smallest distance in both ascending and descending series. Each series was performed twice, and the average of four measurements was used as the representative value of the sensory threshold. Detailed measurement methods for the tactile pressure sensitivity and two-point discrimination are provided below:

Measurement Method for Tactile Pressure Sensitivity

The tactile pressure sensitivity of the plantar surface was assessed using Bell’s method [16], employing 20 types of Semmes-Weinstein monofilaments of varying thicknesses (Sakai Medical Co., Ltd., Sakai City, Japan). The participants were positioned barefoot in the supine position on a bed, and measurements were taken at the big toe, ball of the little toe, and the central heel. The evaluation began with the thinnest filament and progressed sequentially according to thickness. These sites were selected based on previous studies as optimal sites for evaluating plantar tactile pressure sensitivity [17]. Each filament was applied perpendicularly to the skin for 1.5 seconds, exerting pressure until it bent and then withdrawn over another 1.5 seconds. Three stimulations were applied to each site, and if a response was recorded at least once, sensitivity was considered confirmed.

A psychophysical staircase method was used, comprising an ascending series starting from the smallest filament and a descending series beginning with an easily perceptible filament. Each series was conducted twice, and the median of the detectable values was used as the sensory threshold. All measurements were performed by the same evaluator to ensure consistency.

Measurement Method for Two-Point Discrimination

Two-point discrimination of the plantar surface was assessed using a Disk-Criminator (Natsume Seisakusho Co. Ltd., Tokyo, Japan). The participants, positioned barefoot in the supine position on a bed, were assessed at the big toe, ball of the little toe, and heel to determine the minimum distance required to perceive the two distinct points. The minimum distance was defined as the distance at which participants correctly identified two points in at least two of three trials. Participants were instructed to respond “2” if they perceived two points and “1” if they perceived only one.

Prior to measurement, participants were given an orientation to ensure that they could reliably differentiate between one- and two-point contacts [18]. Using the psychophysical staircase method [19], an ascending series began with the smallest distance (at which participants’ responses shifted from one to two points), and a descending series began with an easily detectable distance (where responses shifted from two to one point). Each series was conducted twice, and the average of the four trials was used as the sensory threshold. All assessments were conducted by the same evaluator to ensure consistency.

Statistical analyses

Participant characteristics between groups were analyzed using a one-way analysis of variance (ANOVA) or the chi-square test. To evaluate the effects of the intervention on tactile pressure sensitivity, a Wilcoxon signed-rank test was performed for each group (Control, Discrimination, and Attention) and measurement location (big toe, ball of the little toe, and heel). The median (interquartile range) for each group and location was reported. If no differences were observed between the pre- and post-intervention values, statistical testing was not conducted, and results were noted accordingly.

A two-way repeated-measures ANOVA was conducted to assess two-point discrimination of the foot sole at each measurement site (big toe, ball of the little toe, and heel), based on two factors: "Group" (Discrimination, Attention, and Control groups) and "Intervention Phase" (pre/post). This analysis evaluated the main effects of "Group" and "Intervention Phase," as well as their interaction. All statistical analyses were performed using the R Commander software (version 2.8.1; John Fox, McMaster University, USA), with significance set at 5%.

## Results

No significant differences were observed in baseline patient characteristics between groups (Table 1).

**Table 1:** Basic characteristics of participants in each group SD: standard deviation; BMI: body mass index

Characteristics	Discrimination group (n=11)	Attention group (n=11)	Control group (n=11)	P-value
Age, mean (SD), y	21.00 (0.45)	21.55 (0.82)	20.91 (0.59)	0.10
Height, mean (SD), cm	166.13 (8.30)	165.01 (7.61)	164.27 (9.68)	0.88
Weight, mean (SD), kg	60.05 (7.21)	59.46 (5.75)	60.05 (8.30)	0.98
BMI, mean (SD)	21.68 (0.93)	21.79 (0.39)	22.14 (0.59)	0.28
Gender, woman/man	5/6	6/5	6/5	0.86
Dominant foot, left/right	1/10	2/9	1/10	0.75

In the Discrimination group, no significant changes in tactile pressure sensitivity were noted across measurement locations. Specifically, for the big toe, the test statistic was 0.0, with a p-value of 0.25, indicating no significant intervention effect. Similarly, the ball of the little toe had a test statistic of 0.0 and a p-value of 0.125, while the heel also showed no significant change, with a test statistic of 0.0 and a p-value of 0.5 (Table 2).

**Table 2:** Tactile pressure sensitivity results by group and location Data are presented as median (interquartile range).

	Discrimination group (n=11)		Attention group (n=11)		Control group (n=11)		P-value
	Pre	Post	Pre	Post	Pre	Post	
Big toe (g)	0.07 (0.04-0.4)	0.07 (0.04-0.4)	0.07 (0.02-0.6)	0.07 (0.02-0.6)	0.07 (0.04-0.40)	0.07 (0.04-2.00)	n.s
Ball of the little toe (g)	0.07 (0.04-0.16)	0.07 (0.04-0.16)	0.16 (0.04-0.16)	0.16 (0.04-0.4)	0.04 (0.04-0.16)	0.04 (0.04-0.16)	n.s
Heel (g)	2.00 (0.04-2.00)	2.00 (0.04-2.00)	2.00 (0.04-2.00)	2.00 (0.04-2.00)	2.00 (0.04-2.00)	2.00 (0.04-2.00)	n.s

In the Attention group, no measurable changes were observed in the big toe or heel between pre- and post-intervention values, so statistical tests were not conducted for these locations. For the ball of the little toe, the test statistic was 4.5, with a p-value of 0.875, indicating no significant differences (Table 2).

In the Control group, tactile pressure sensitivity remained stable across all measurement locations. Both the big toe and ball of the little toe showed a test statistic of 0.0 with p-values of 0.25, indicating no significant difference between the pre- and post-intervention measurements. The heel also showed no significant change, with a test statistic of 0.0 and a p-value of 0.5 (Table 2).

The main effect of the Intervention Phase was statistically significant (F (1, 10) = 21.78, p = 0.0009). However, the main effect of the group was not significant (F (2,20) = 2.22, p= 0.1349). A significant interaction effect between the Group and Intervention Phase was observed (F (2, 20) = 16.13, p = 0.0001). Given the significant interaction, post hoc comparisons using Tukey's Honest Significant Difference (HSD) test showed that the discrimination group exhibited a significantly greater change from pre- to post-intervention than the control and attention groups (Table 3).

**Table 3:** Analysis results of two-point discrimination by plantar region

	Discrimination group (n=11)		Attention group (n=11)		Control group (n=11)		Main effect (Group)		Main effect (Intervention)		Interaction effect		Significant post-hoc comparisons
	Pre	Post	Pre	Post	Pre	Post	F-value	P-value	F-value	P-value	F-value	P-value	
Big toe (mm)	8.55 ± 1.92	6.45 ± 1.63	8.73 ± 1.42	8.55 ± 1.92	8.82 ± 1.40	8.55 ± 1.37	2.22	0.13	21.78	<0.001	16.13	<0.001	Discrimination_Pre vs Discrimination_Post (<0.05)
Ball of the little toe (mm)	9.36 ± 1.75	8.36 ± 2.01	10.18 ± 2.99	10.55 ± 1.63	10.45 ± 2.58	9.45 ± 2.73	1.71	0.21	8.53	0.02	2.90	0.08	n.s
Heel (mm)	10.45 ± 2.77	10.09 ± 2.63	10.82 ± 3.37	10.45 ± 2.16	11.73 ± 1.74	11.82 ± 1.66	1.14	0.34	0.65	0.44	0.52	0.60	n.s

A significant main effect of the Intervention Phase was also found for the ball of the little toe (F (1, 10) =8.53, p=0.0153), indicating a general effect of the intervention. However, neither the main effect of Group F (2, 20) = 1.71, p=0.2060 nor the interaction effect between Group and Intervention Phase F (2, 20) =2.90, p=0.0784 reached significance, so no post-hoc analysis was conducted for this measurement site (Table 3).

For the heel measurement, neither the main effect of the Intervention Phase F (1, 10) = 0.65, p=0.4389, p = 0.4389 nor the main effect of Group F (2,20) =1.14, p=0.3399 were significant. Additionally, the interaction effect between the Group and Intervention Phase was not significant, F (2, 20) = 0.52, p=0.6025, so no further post hoc analysis was conducted for the heel (Table 3).

## Discussion

In this study, we examined the effects of foot somatosensory training on tactile pressure sensitivity and two-point discrimination of the foot sole. Results indicated no significant changes in tactile pressure sensitivity across any group. However, significant improvements in two-point discrimination were observed in the group that underwent discrimination training, specifically in the big toe. These findings suggest that foot somatosensory training may be effective in enhancing two-point discrimination, particularly in the big toe. Here, we discuss the characteristics of tactile pressure sensitivity and two-point discrimination, task condition differences, the influence of two-point discrimination on higher cortical areas, and specific effects observed in the big toe.

Differences between plantar tactile pressure sensitivity and two-point discrimination

Plantar tactile pressure sensitivity and two-point discrimination are both used to assess somatosensory function; however, they differ in sensory characteristics and processing mechanisms. Plantar tactile pressure sensitivity refers to the ability to detect pressure intensity or contact on the skin, primarily involving low-threshold mechanoreceptors, such as Merkel disks and Pacinian corpuscles [20]. These receptors perform simple processing to detect pressure changes, thus requiring minimal spatial cognitive processing and exerting a limited influence on higher brain regions.

In contrast, plantar two-point discrimination is a complex sensation that requires spatial discrimination abilities involving Merkel disks and Ruffini endings [21]. Additionally, two-point discrimination has long been studied as an indicator of spatial resolution of tactile pressure sensitivity and is considered to reflect spatial resolution within somatosensory function [22,23]. The processing of　two-point discrimination involves not only the primary somatosensory cortex (S1) but also the secondary somatosensory cortex (S2) located in the parietal lobe, as well as the posterior parietal cortex (PPC), a higher-order region responsible for spatial and cognitive processing [24]. These cortical areas are known to exhibit neuroplastic changes in response to sensory training, resulting in improved sensory map precision and enhanced differentiation of closely spaced stimuli. Discrimination tasks, by repeatedly engaging these neural circuits, may strengthen synaptic connections and refine sensory processing mechanisms, contributing to the observed improvements in two-point discrimination.

Differences in conditions: Discrimination training, attention group, and control group

Each group - Discrimination, Attention, and Control - received similar plantar somatosensory input but engaged with it differently.

The Discrimination group performed tasks requiring “spatial attention” to sensory stimuli by identifying the location of weights applied to the plantar surface. This task likely activated not only S1 but also S2 and PPC, areas involved in spatial discrimination, attention processing, higher-order spatial cognition, and sensory integration. This spatially attentive task may have led to neuroplastic changes in S2 and PPC, resulting in the reorganization of sensory maps and a significant improvement in two-point discrimination.

In contrast, the Attention group was tasked with focusing on the “timing” of sensory input without spatial discrimination demands. The task involving quick responses emphasized cognitive processes such as sustained attention and temporal precision. These aspects are known to engage neural networks in the frontal and parietal cortices, critical for attentional focus and sensory integration [14,15]. Although the attention task did not involve spatial discrimination, it effectively required participants to maintain high levels of focus and attentional engagement, contributing to the observed differences between groups. Although the sensory input engaged S1, the lack of spatial processing indicated that S2 and PPC involvement were limited. Consequently, this group showed no improvement in two-point discrimination, suggesting that spatial cognitive engagement is essential for enhancing this function.

Finally, in the Control group, participants received plantar somatosensory input while attending to a separate visual task that minimized cognitive involvement with plantar sensory input. As a result, there was insufficient sensory processing in S1 and S2, leading to no improvement in two-point discrimination. These findings suggest that spatially attentive discrimination training, rather than sensory stimulation alone, is crucial for improving two-point discrimination in the plantar region.

Consideration of effects observed in the big toe

Improvement in plantar two-point discrimination was only observed in the big toe. The big toe plays a key role in maintaining balance during standing and stability while walking, emphasizing its importance in sensory function [25-27]. The big toe has a high density of Merkel disks and Ruffini endings, which are specialized for efficient sensory processing and possess a well-developed neural structure that supports this function. Additionally, the big toe has a particularly large cortical representation in the sensory cortex [28], making it a site where the neural activity in the S1, S2, and PPC is more easily activated. This may explain the pronounced improvement in two-point discrimination in the big toe compared to other areas of the foot sole, highlighting its distinct responsiveness to sensory training. In contrast, the ball of the little toe and the central heel did not show significant improvements in two-point discrimination. These areas have a lower density of mechanoreceptors, such as Merkel disks and Ruffini endings, compared to the big toe, which may limit their capacity for sensory differentiation. Moreover, the cortical representation of these regions in the somatosensory cortex is relatively smaller, reducing their potential for neural plasticity and sensory map refinement. Additionally, the functional demands placed on these areas during daily activities, such as walking or balance maintenance, are lower compared to the big toe, which could contribute to their limited responsiveness to sensory training.

Absence of change in plantar tactile pressure sensitivity

No significant changes in plantar tactile pressure sensitivity were observed in any group. This may be because tactile pressure sensitivity, a simple sensation specialized for pressure detection, does not require spatial cognitive processing. The discrimination tasks used in this study involved spatial cognitive processing, engaging higher brain areas such as S2 and PPC, making them less likely to impact basic sensory processing, such as tactile pressure sensitivity. This suggests that improving tactile pressure sensitivity may require training focused specifically on pressure perception.

Clinical implications and future applications

This study’s findings suggest that discrimination tasks aimed at enhancing spatial discrimination abilities are effective for foot somatosensory training, particularly in improving two-point discrimination in the big toe. Improvements in two-point discrimination in the big toe may directly enhance balance and stability during walking, highlighting the clinical significance of this approach. Such training could improve daily living activities and help prevent falls in elderly individuals and patients with sensory impairments. To incorporate this training into a therapeutic context, therapists could design structured programs that use similar discrimination tasks tailored to individual needs. For example, tasks could be adapted for seated or standing positions, depending on the patient’s balance and mobility levels. Additionally, the use of low-cost equipment, such as instability boards or simple weight placement setups, would make this approach accessible in clinical and home-based settings. Future studies should investigate the optimal frequency, duration, and intensity of such training to maximize its clinical benefits. Conversely, improving tactile pressure sensitivity may require alternative approaches specifically tailored to pressure detection.

Study limitations

Limitations of this study include a relatively small sample size. While immediate intervention effects were demonstrated, long-term effects and applicability to older individuals and those with brain injuries remain unexplored. Additionally, the study only included healthy young adults, which limits the generalizability of the findings to older adults and individuals with neurological conditions. These populations are at greater risk for sensory impairments, and further studies are necessary to validate the clinical relevance of these results in such groups. Future research should assess whether the long-term effects of the intervention tasks employed in this study and their efficacy in older adults or neurologically impaired populations are comparable to those observed in healthy young adults. Furthermore, this study did not evaluate the proprioceptive function of the ankle, which is essential for controlling an unstable board to accurately perceive weight. Future studies should involve larger samples to validate and expand on these findings. Moreover, this study did not account for potential confounding variables such as participants' physical activity levels or any history of sensory impairments. These factors may influence sensory function and training responsiveness, potentially affecting the observed outcomes. Future studies should carefully control these variables to ensure more accurate interpretations of the results and their applicability to diverse populations.

## Conclusions

This study suggests that active somatosensory discrimination tasks may serve as an effective therapeutic approach for patients with sensory impairments, given the lack of established physical therapy interventions targeting sensory function enhancement. The findings demonstrate that foot somatosensory training can reduce the two-point discrimination threshold in healthy individuals without plantar sensory dysfunction, thereby enhancing two-point discrimination sensitivity. This is the first study to document changes in plantar somatosensory function, specifically in tactile pressure sensitivity and two-point discrimination thresholds, following foot sensory training. These results provide valuable foundational data for clinical applications of sensory-function-focused physical therapy and underscore its potential for rehabilitation. Future studies with larger, more diverse samples, including elderly and neurologically impaired populations, are warranted to further validate these findings and assess the long-term efficacy of foot somatosensory training.
